# Tetra­kis[μ_3_-4-nitro-*N*-(5-phenyl-1,3,4-oxadiazol-2-yl)benzamidato]tetra­kis­[methano­lsodium(I)]

**DOI:** 10.1107/S1600536812014791

**Published:** 2012-04-13

**Authors:** Guo-Jie Yin, Qing Zhang, Dong Li

**Affiliations:** a Department of Environment Engineering and Chemistry, Luoyang Institute of Science and Technology, 471023 Luoyang, People’s Republic of China

## Abstract

In the title compound, [Na_4_(C_15_H_9_N_4_O_4_)_4_(CH_3_OH)_4_], the N_3_O_3_ environment around the Na^+^ ion is distorted octa­hedral. In the unit cell, four Na^+^ ions are bridged by four Schiff base anions, leading to a tetra­nuclear complex with -4 symmetry. O—H⋯N hydrogen bonds between the methanol mol­ecule and the Schiff base anion stabilize the structural set-up.

## Related literature

For the preparation of 2-amino-5-phenyl-1,3,4-oxadiazole, see: Gibson (1962 ▶) and of *N*-(5-phenyl-1,3,4-oxadiazol-2-yl)-*p*-nitro­benzamide, see: Zhang *et al.* (2009 ▶). Organic ligands based on oxadiazole or carboxyl­ate groups have both good coordination ability and diverse coordination modes, see: Hu *et al.* (2008 ▶).
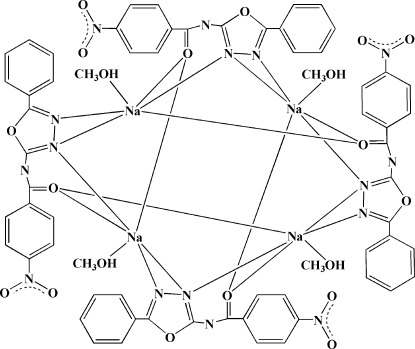


## Experimental

### 

#### Crystal data

[Na_4_(C_15_H_9_N_4_O_4_)_4_(CH_4_O)_4_]
*M* *_r_* = 1457.18
Tetragonal, 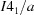

*a* = 15.6635 (2) Å
*c* = 27.1833 (6) Å
*V* = 6669.29 (18) Å^3^
*Z* = 4
Mo *K*α radiation
μ = 0.13 mm^−1^
*T* = 293 K
0.30 × 0.20 × 0.20 mm

#### Data collection

Bruker SMART CCD area-detector diffractometer
Absorption correction: multi-scan (*SADABS*; Sheldrick, 2004 ▶) *T*_min_ = 0.934, *T*_max_ = 1.000
15582 measured reflections
3413 independent reflections
2653 reflections with *I* > 2σ(*I*)
*R*_int_ = 0.027

#### Refinement

*R*[*F* ^2^ > 2σ(*F* ^2^)] = 0.040
*wR*(*F* ^2^) = 0.106
*S* = 1.02
3413 reflections
239 parameters
2 restraints
H atoms treated by a mixture of independent and constrained refinement
Δρ_max_ = 0.22 e Å^−3^
Δρ_min_ = −0.22 e Å^−3^

### 

Data collection: *SMART* (Bruker, 2001 ▶); cell refinement: *SAINT* (Bruker, 2001 ▶); data reduction: *SAINT*; program(s) used to solve structure: *SHELXS97* (Sheldrick, 2008 ▶); program(s) used to refine structure: *SHELXL97* (Sheldrick, 2008 ▶); molecular graphics: *SHELXTL* (Sheldrick, 2008 ▶); software used to prepare material for publication: *SHELXL97*.

## Supplementary Material

Crystal structure: contains datablock(s) global, I. DOI: 10.1107/S1600536812014791/hp2035sup1.cif

Structure factors: contains datablock(s) I. DOI: 10.1107/S1600536812014791/hp2035Isup2.hkl

Additional supplementary materials:  crystallographic information; 3D view; checkCIF report

## Figures and Tables

**Table 1:** Selected bond lengths (Å)

Na1—O5	2.3413 (14)
Na1—N1	2.3828 (15)
Na1—O1	2.3848 (13)
Na1—O1^i^	2.3972 (13)
Na1—N2^ii^	2.4127 (15)
Na1—N1^ii^	2.9859 (15)

Symmetry codes: (i) 
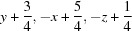
; (ii) 
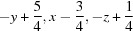
.

**Fig. 1.**
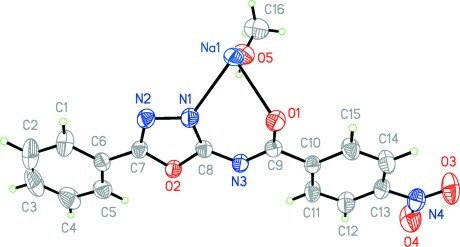
The molecular structure of the title complex with displacement ellipsoids drawn at the 50% probability level.

**Fig. 2.**
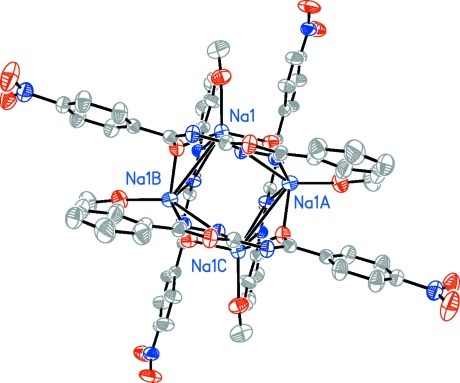
The molecular structure of the title complex with displacement ellipsoids drawn at the 50% probability level. Hydrogen atoms are omitted for clarity.

## supplementary crystallographic information

### Comment

As we all know, the organic ligands based on oxadiazole or carboxylate groups which containing N and O donors have both good coordination ability and diverse coordination modes (Hu *et al.*, 2008). Therefore, the ligand *N*-(5-phenyl-1,3,4-oxadiazol-2-yl)- *p*-nitrobenzamide was chosen to create coordination architectures.

In the title compound, each Na^I^ atom is six-coordinated by one O atom from a methyl alcohol, two O atoms and three N atoms from the ligands, forming a distorted octahedral geometry. In the asymmetric unit, the four Na^I^ ions are bridged by four Schiff base anions, leading to a tetranuclear complex (Fig. 1 and Fig. 2), the coordination geometry of sodium ions can be described as distorted quadrilateral.

### Experimental

Reagents and solvents were of commercially available quality. The preparation of 2-Amino-5-phenyl-1,3,4-oxadiazole is based on a published method (Gibson, 1962). Bromine (0.66 ml) in glacial acetic acid (1.34 ml) was added to a stirred slurry of benzaldehyde semicarbazone (2.0 g) and powdered, anhydrous sodium acetate (4.0 g) in acetic acid (12 ml). The solids were dissolved giving a red solution, which suddenly grew warm and rapidly faded with white precipitate formed (sodium bromide). After 15 minutes, the mixture was poured into water (100 ml), and the precipitated solid (1.8 g) collected, washed and dried. Crystallization from ethanol gave stout needles.

The ligand of *N*-(5-phenyl-1,3,4-oxadiazol-2-yl)-*p*-nitrobenzamide was synthesized according to the method of literature (Zhang *et al.*, 2009). 4-Nitrobenzoyl chloride (5.94 g, 0.032 mol) was dropped slowly into the stirred slurry of 2-Amino-5-phenyl-1,3,4-oxadiazole (5.64 g, 0.035 mol) in 50 ml pyridine. 2-Amino-5-phenyl-1,3,4-oxadiazole dissolved gradually and gave a buff solution. After 2 h, the solution was poured into water. Then the sodium hydroxide (2.80 g, 0.07 mol) was added to give a alkaline solution, the precipitate was collected and dried under vacuum. The title compound was obtained by re-crystallization from ethanol. Yield: 5.7 g, 60%.

### Refinement

H atoms were positioned geometrically and refined using a riding model with C—H =0.93–0.96 Å and with *U*_iso_(H) = 1.2 (1.5 for methyl groups) times *U*_eq_(C).

### Crystal data

**Table:**

[Na_4_(C_15_H_9_N_4_O_4_)_4_(CH_4_O)_4_]	*D*_x_ = 1.451 Mg m^−^^3^
*M**_r_* = 1457.18	Mo *K*α radiation, λ = 0.71073 Å
Tetragonal, *I*4_1_/*a*	Cell parameters from 5181 reflections
*a* = 15.6635 (2) Å	θ = 3.0–29.0°
*c* = 27.1833 (6) Å	µ = 0.13 mm^−^^1^
*V* = 6669.29 (18) Å^3^	*T* = 293 K
*Z* = 4	Prismatic, yellow
*F*(000) = 3008	0.30 × 0.20 × 0.20 mm

### Data collection

**Table:**

Bruker SMART CCD area-detector diffractometer	3413 independent reflections
Radiation source: fine-focus sealed tube	2653 reflections with *I* > 2σ(*I*)
Graphite monochromator	*R*_int_ = 0.027
phi and ω scans	θ_max_ = 26.4°, θ_min_ = 3.0°
Absorption correction: multi-scan (*SADABS*; Sheldrick, 2004)	*h* = −17→19
*T*_min_ = 0.934, *T*_max_ = 1.000	*k* = −19→19
15582 measured reflections	*l* = −33→30

### Refinement

**Table:**

Refinement on *F*^2^	Primary atom site location: structure-invariant direct methods
Least-squares matrix: full	Secondary atom site location: difference Fourier map
*R*[*F*^2^ > 2σ(*F*^2^)] = 0.040	Hydrogen site location: inferred from neighbouring sites
*wR*(*F*^2^) = 0.106	H atoms treated by a mixture of independent and constrained refinement
*S* = 1.02	*w* = 1/[σ^2^(*F*_o_^2^) + (0.0488*P*)^2^ + 3.8193*P*] where *P* = (*F*_o_^2^ + 2*F*_c_^2^)/3
3413 reflections	(Δ/σ)_max_ < 0.001
239 parameters	Δρ_max_ = 0.22 e Å^−^^3^
2 restraints	Δρ_min_ = −0.22 e Å^−^^3^

### Special details

**Table:**

Geometry. All e.s.d.'s (except the e.s.d. in the dihedral angle between two l.s. planes) are estimated using the full covariance matrix. The cell e.s.d.'s are taken into account individually in the estimation of e.s.d.'s in distances, angles and torsion angles; correlations between e.s.d.'s in cell parameters are only used when they are defined by crystal symmetry. An approximate (isotropic) treatment of cell e.s.d.'s is used for estimating e.s.d.'s involving l.s. planes.
Refinement. Refinement of *F*^2^ against ALL reflections. The weighted *R*-factor *wR* and goodness of fit *S* are based on *F*^2^, conventional *R*-factors *R* are based on *F*, with *F* set to zero for negative *F*^2^. The threshold expression of *F*^2^ > σ(*F*^2^) is used only for calculating *R*-factors(gt) *etc*. and is not relevant to the choice of reflections for refinement. *R*-factors based on *F*^2^ are statistically about twice as large as those based on *F*, and *R*- factors based on ALL data will be even larger.

### Fractional atomic coordinates and isotropic or equivalent isotropic displacement parameters (Å^2^)

**Table:**

	*x*	*y*	*z*	*U*_iso_*/*U*_eq_	
Na1	0.83748 (4)	0.26324 (4)	0.11911 (2)	0.03798 (19)	
O1	0.85910 (8)	0.13991 (7)	0.16902 (4)	0.0404 (3)	
O2	0.90153 (7)	0.31903 (7)	0.27519 (4)	0.0363 (3)	
O3	0.65522 (12)	−0.23034 (10)	0.22897 (7)	0.0783 (5)	
O4	0.63373 (12)	−0.18639 (10)	0.30241 (6)	0.0805 (5)	
O5	0.69874 (9)	0.29271 (10)	0.14628 (5)	0.0532 (4)	
H5	0.6938 (13)	0.2932 (16)	0.1773 (3)	0.080*	
N1	0.90347 (9)	0.30288 (9)	0.19472 (5)	0.0386 (3)	
N2	0.93939 (9)	0.38225 (9)	0.20680 (5)	0.0388 (3)	
N3	0.84467 (9)	0.19219 (8)	0.24835 (5)	0.0352 (3)	
C1	1.00589 (13)	0.52826 (12)	0.26187 (9)	0.0571 (5)	
H1	1.0161	0.5274	0.2282	0.069*	
C2	1.03166 (15)	0.59702 (14)	0.28927 (12)	0.0733 (7)	
H2	1.0593	0.6426	0.2742	0.088*	
C3	1.01644 (15)	0.59842 (15)	0.33943 (12)	0.0743 (8)	
H3	1.0341	0.6448	0.3582	0.089*	
C4	0.97544 (15)	0.53153 (15)	0.36130 (9)	0.0660 (6)	
H4	0.9649	0.5329	0.3950	0.079*	
C5	0.94954 (13)	0.46210 (12)	0.33403 (7)	0.0488 (5)	
H5A	0.9219	0.4167	0.3492	0.059*	
C6	0.96483 (10)	0.46020 (10)	0.28401 (7)	0.0373 (4)	
C7	0.93688 (10)	0.38873 (10)	0.25373 (6)	0.0330 (4)	
C8	0.88199 (10)	0.26667 (10)	0.23605 (6)	0.0324 (4)	
C9	0.83531 (10)	0.13374 (10)	0.21273 (6)	0.0322 (4)	
C10	0.79134 (10)	0.05235 (10)	0.22781 (6)	0.0332 (4)	
C11	0.76375 (13)	0.03611 (11)	0.27529 (7)	0.0468 (5)	
H11	0.7734	0.0763	0.2998	0.056*	
C12	0.72214 (13)	−0.03911 (12)	0.28662 (7)	0.0504 (5)	
H12	0.7033	−0.0496	0.3185	0.060*	
C13	0.70909 (11)	−0.09794 (10)	0.25015 (7)	0.0409 (4)	
C14	0.73597 (13)	−0.08450 (12)	0.20282 (7)	0.0503 (5)	
H14	0.7268	−0.1254	0.1786	0.060*	
C15	0.77700 (12)	−0.00895 (12)	0.19195 (7)	0.0460 (4)	
H15	0.7954	0.0011	0.1599	0.055*	
N4	0.66286 (11)	−0.17717 (10)	0.26138 (7)	0.0527 (4)	
C16	0.61860 (15)	0.30244 (17)	0.12369 (9)	0.0732 (7)	
H16A	0.6260	0.3257	0.0913	0.110*	
H16B	0.5840	0.3404	0.1429	0.110*	
H16C	0.5911	0.2478	0.1214	0.110*	

### Atomic displacement parameters (Å^2^)

**Table:**

	*U*^11^	*U*^22^	*U*^33^	*U*^12^	*U*^13^	*U*^23^
Na1	0.0474 (4)	0.0400 (4)	0.0266 (3)	0.0005 (3)	0.0019 (3)	−0.0022 (3)
O1	0.0541 (8)	0.0366 (6)	0.0305 (6)	−0.0014 (5)	0.0096 (5)	−0.0032 (5)
O2	0.0489 (7)	0.0342 (6)	0.0257 (6)	−0.0075 (5)	0.0036 (5)	−0.0010 (5)
O3	0.0961 (13)	0.0499 (9)	0.0890 (12)	−0.0320 (8)	0.0193 (10)	−0.0198 (9)
O4	0.1073 (14)	0.0652 (10)	0.0691 (11)	−0.0349 (9)	0.0210 (10)	0.0069 (8)
O5	0.0461 (8)	0.0717 (9)	0.0420 (7)	−0.0027 (7)	0.0096 (6)	−0.0025 (7)
N1	0.0496 (9)	0.0373 (8)	0.0289 (7)	−0.0089 (7)	0.0050 (6)	−0.0008 (6)
N2	0.0448 (8)	0.0390 (8)	0.0327 (8)	−0.0083 (6)	0.0054 (6)	0.0014 (6)
N3	0.0436 (8)	0.0327 (7)	0.0293 (7)	−0.0048 (6)	0.0049 (6)	−0.0016 (6)
C1	0.0543 (12)	0.0471 (11)	0.0699 (14)	−0.0149 (9)	0.0119 (10)	−0.0055 (10)
C2	0.0597 (14)	0.0465 (13)	0.114 (2)	−0.0196 (10)	0.0090 (14)	−0.0121 (13)
C3	0.0555 (13)	0.0551 (14)	0.112 (2)	−0.0034 (11)	−0.0177 (14)	−0.0392 (14)
C4	0.0749 (16)	0.0620 (14)	0.0611 (14)	0.0022 (12)	−0.0168 (12)	−0.0249 (12)
C5	0.0574 (12)	0.0459 (11)	0.0431 (10)	0.0000 (9)	−0.0065 (9)	−0.0064 (9)
C6	0.0322 (9)	0.0350 (9)	0.0445 (10)	−0.0005 (7)	−0.0022 (7)	−0.0039 (8)
C7	0.0323 (8)	0.0330 (8)	0.0337 (9)	−0.0034 (7)	0.0030 (7)	0.0021 (7)
C8	0.0357 (8)	0.0348 (8)	0.0267 (8)	−0.0022 (7)	0.0034 (7)	−0.0036 (7)
C9	0.0332 (8)	0.0324 (8)	0.0310 (8)	0.0029 (7)	0.0020 (7)	−0.0014 (7)
C10	0.0333 (8)	0.0315 (8)	0.0347 (8)	0.0013 (7)	0.0013 (7)	−0.0026 (7)
C11	0.0656 (12)	0.0363 (9)	0.0384 (10)	−0.0087 (8)	0.0096 (9)	−0.0070 (8)
C12	0.0681 (13)	0.0432 (10)	0.0399 (10)	−0.0099 (9)	0.0136 (9)	0.0003 (8)
C13	0.0398 (9)	0.0330 (9)	0.0498 (10)	−0.0038 (7)	0.0046 (8)	0.0003 (8)
C14	0.0611 (12)	0.0421 (10)	0.0476 (11)	−0.0128 (9)	0.0047 (9)	−0.0114 (9)
C15	0.0571 (12)	0.0445 (10)	0.0365 (9)	−0.0103 (9)	0.0068 (8)	−0.0058 (8)
N4	0.0525 (10)	0.0408 (9)	0.0649 (12)	−0.0084 (7)	0.0070 (9)	0.0005 (9)
C16	0.0564 (14)	0.0979 (19)	0.0654 (15)	−0.0038 (12)	0.0021 (12)	0.0079 (14)

### Geometric parameters (Å, º)

**Table:**

Na1—O5	2.3413 (14)	C1—H1	0.9300
Na1—N1	2.3828 (15)	C2—C3	1.384 (4)
Na1—O1	2.3848 (13)	C2—H2	0.9300
Na1—O1^i^	2.3972 (13)	C3—C4	1.365 (4)
Na1—N2^ii^	2.4127 (15)	C3—H3	0.9300
Na1—N1^ii^	2.9859 (15)	C4—C5	1.377 (3)
Na1—Na1^i^	3.6261 (9)	C4—H4	0.9300
Na1—Na1^ii^	3.6261 (9)	C5—C6	1.381 (3)
O1—C9	1.2491 (19)	C5—H5A	0.9300
O1—Na1^ii^	2.3972 (13)	C6—C7	1.457 (2)
O2—C7	1.3559 (19)	C9—C10	1.506 (2)
O2—C8	1.3778 (18)	C10—C11	1.385 (2)
O3—N4	1.218 (2)	C10—C15	1.387 (2)
O4—N4	1.214 (2)	C11—C12	1.381 (3)
O5—C16	1.406 (3)	C11—H11	0.9300
O5—H5	0.847 (9)	C12—C13	1.369 (3)
N1—C8	1.303 (2)	C12—H12	0.9300
N1—N2	1.4036 (19)	C13—C14	1.370 (3)
N1—Na1^i^	2.9859 (15)	C13—N4	1.469 (2)
N2—C7	1.280 (2)	C14—C15	1.379 (3)
N2—Na1^i^	2.4127 (15)	C14—H14	0.9300
N3—C9	1.341 (2)	C15—H15	0.9300
N3—C8	1.347 (2)	C16—H16A	0.9600
C1—C2	1.370 (3)	C16—H16B	0.9600
C1—C6	1.383 (3)	C16—H16C	0.9600
			
O5—Na1—N1	94.54 (5)	C3—C2—H2	120.1
O5—Na1—O1	96.43 (5)	C4—C3—C2	119.8 (2)
N1—Na1—O1	70.05 (5)	C4—C3—H3	120.1
O5—Na1—O1^i^	106.86 (5)	C2—C3—H3	120.1
N1—Na1—O1^i^	90.40 (5)	C3—C4—C5	120.7 (2)
O1—Na1—O1^i^	150.81 (5)	C3—C4—H4	119.7
O5—Na1—N2^ii^	123.14 (5)	C5—C4—H4	119.7
N1—Na1—N2^ii^	141.02 (6)	C4—C5—C6	119.7 (2)
O1—Na1—N2^ii^	94.03 (5)	C4—C5—H5A	120.1
O1^i^—Na1—N2^ii^	87.87 (5)	C6—C5—H5A	120.1
O5—Na1—N1^ii^	146.17 (5)	C5—C6—C1	119.51 (17)
N1—Na1—N1^ii^	113.69 (6)	C5—C6—C7	121.38 (16)
O1—Na1—N1^ii^	77.43 (4)	C1—C6—C7	119.10 (17)
O1^i^—Na1—N1^ii^	91.84 (4)	N2—C7—O2	112.17 (14)
N2^ii^—Na1—N1^ii^	27.62 (4)	N2—C7—C6	127.92 (15)
O5—Na1—Na1^i^	123.35 (5)	O2—C7—C6	119.89 (14)
N1—Na1—Na1^i^	55.00 (4)	N1—C8—N3	134.68 (15)
O1—Na1—Na1^i^	111.36 (4)	N1—C8—O2	110.45 (13)
O1^i^—Na1—Na1^i^	40.56 (3)	N3—C8—O2	114.86 (13)
N2^ii^—Na1—Na1^i^	103.62 (4)	O1—C9—N3	126.97 (15)
N1^ii^—Na1—Na1^i^	89.20 (3)	O1—C9—C10	117.45 (14)
O5—Na1—Na1^ii^	136.08 (5)	N3—C9—C10	115.58 (14)
N1—Na1—Na1^ii^	80.91 (4)	C11—C10—C15	118.53 (16)
O1—Na1—Na1^ii^	40.82 (3)	C11—C10—C9	123.50 (15)
O1^i^—Na1—Na1^ii^	116.78 (3)	C15—C10—C9	117.97 (15)
N2^ii^—Na1—Na1^ii^	65.47 (4)	C12—C11—C10	120.78 (17)
N1^ii^—Na1—Na1^ii^	40.82 (3)	C12—C11—H11	119.6
Na1^i^—Na1—Na1^ii^	89.553 (3)	C10—C11—H11	119.6
C9—O1—Na1	124.17 (10)	C13—C12—C11	118.90 (17)
C9—O1—Na1^ii^	129.32 (11)	C13—C12—H12	120.5
Na1—O1—Na1^ii^	98.63 (5)	C11—C12—H12	120.5
C7—O2—C8	103.76 (12)	C12—C13—C14	122.05 (16)
C16—O5—Na1	135.43 (13)	C12—C13—N4	119.46 (17)
C16—O5—H5	110.6 (14)	C14—C13—N4	118.47 (16)
Na1—O5—H5	113.6 (14)	C13—C14—C15	118.46 (17)
C8—N1—N2	106.69 (13)	C13—C14—H14	120.8
C8—N1—Na1	121.22 (11)	C15—C14—H14	120.8
N2—N1—Na1	127.35 (10)	C14—C15—C10	121.27 (17)
C8—N1—Na1^i^	154.19 (11)	C14—C15—H15	119.4
N2—N1—Na1^i^	52.83 (7)	C10—C15—H15	119.4
Na1—N1—Na1^i^	84.17 (4)	O4—N4—O3	123.11 (17)
C7—N2—N1	106.93 (13)	O4—N4—C13	118.49 (17)
C7—N2—Na1^i^	148.36 (12)	O3—N4—C13	118.39 (17)
N1—N2—Na1^i^	99.56 (9)	O5—C16—H16A	109.5
C9—N3—C8	117.38 (13)	O5—C16—H16B	109.5
C2—C1—C6	120.4 (2)	H16A—C16—H16B	109.5
C2—C1—H1	119.8	O5—C16—H16C	109.5
C6—C1—H1	119.8	H16A—C16—H16C	109.5
C1—C2—C3	119.8 (2)	H16B—C16—H16C	109.5
C1—C2—H2	120.1		

Symmetry codes: (i) *y*+3/4, −*x*+5/4, −*z*+1/4; (ii) −*y*+5/4, *x*−3/4, −*z*+1/4.

### Hydrogen-bond geometry (Å, º)

**Table:**

*D*—H···*A*	*D*—H	H···*A*	*D*···*A*	*D*—H···*A*
O5—H5···N3^iii^	0.85 (1)	2.12 (1)	2.9534 (19)	167 (2)

Symmetry code: (iii) −*x*+3/2, −*y*+1/2, −*z*+1/2.
